# Plant ribosomes as a score to fathom the melody of 2’-*O*-methylation across evolution

**DOI:** 10.1080/15476286.2024.2417152

**Published:** 2024-11-07

**Authors:** Sara Alina Neumann, Christine Gaspin, Julio Sáez-Vásquez

**Affiliations:** a CNRS, Laboratoire Génome et Développement des Plantes (LGDP), UMR 5096, Perpignan, France; b University Perpignan Via Domitia, LGDP, UMR 5096, Perpignan, France; c Université Fédérale de Toulouse, INRAE, MIAT, Castanet-Tolosan, France; d Université Fédérale de Toulouse, INRAE, BioinfOmics, Genotoul Bioinformatics Facility, Castanet-Tolosan, France

**Keywords:** 2’-*O*-methylation, ribosomal RNA, plants, epitranscriptome, C/D-box snoRNP

## Abstract

2’-*O*-ribose methylation (2’-*O*-Me) is one of the most common RNA modifications detected in ribosomal RNAs (rRNA) from bacteria to eukaryotic cells. 2’-*O*-Me favours a specific RNA conformation and protects RNA from hydrolysis. Moreover, rRNA 2’-*O*-Me might stabilize its interactions with messenger RNA (mRNA), transfer RNA (tRNA) or proteins. The extent of rRNA 2’-*O*-Me fluctuates between species from 3–4 sites in bacteria to tens of sites in archaea, yeast, algae, plants and human. Depending on the organism as well as the rRNA targeting site and position, the 2’-*O*-Me reaction can be carried out by several site-specific RNA methyltransferases (RMTase) or by a single RMTase associated to specific RNA guides. Here, we review current progresses in rRNA 2’-*O*-Me (sites/Nm and RMTases) in plants and compare the results with molecular clues from unicellular (bacteria, archaea, algae and yeast) as well as multicellular (human and plants) organisms.

## 2’-O-ribose methylation and its biochemical consequences

**Figure 1.**
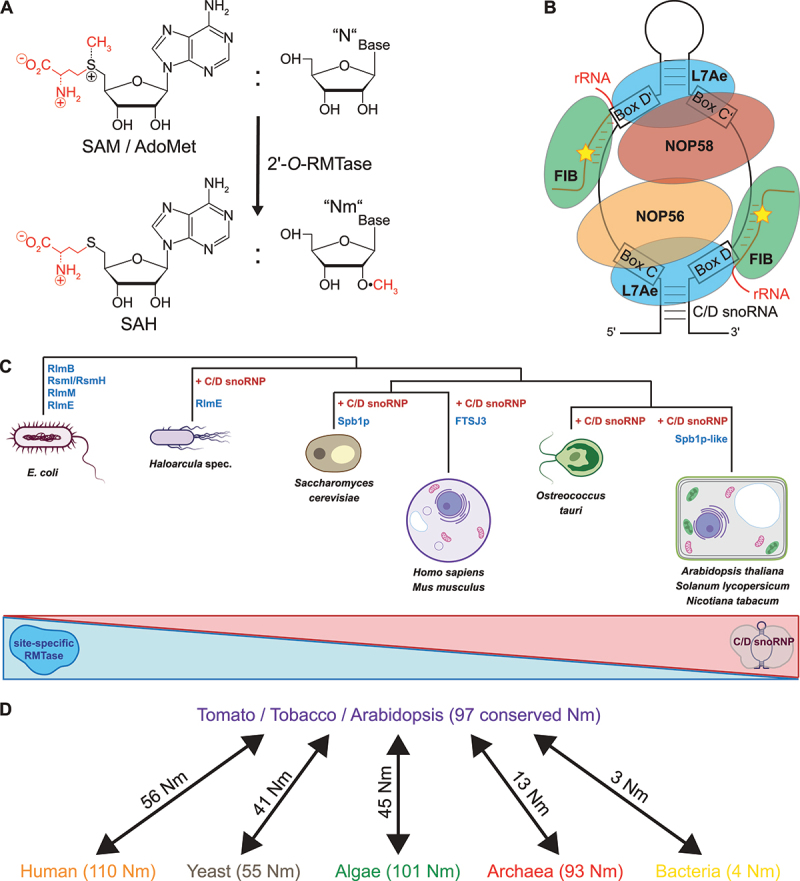
rRNA 2’-*O*-methylation and phylogenetic conservation of RMTase. a) Methyl transfer reaction by RMTase from *S*-adenosylmethionine (SAM/AdoMet) to nucleophiles results in 2’-*O*-methylated RNA nucleotides and *S*-adenosylhomocysteine (SAH). b) Schematic representation of the mammalian C/D snoRNP complex with fibrillarin (FIB, green ellipse), NOP56 (red ellipse), NOP58 (Orange ellipse), L7Ae (blue ellipse) and C/D snoRNA (black line, conserved C/D and less conserved C’/D’ boxes in black rectangles) interacting with the to be methylated site (yellow star) on the rRNA (red line). c) Top, Phylogenetic tree as a representation of the distribution of RMTases as stand-alone enzymes (blue) or forming C/D snoRNP complexes (red) in various species indicated by their latin names and a schematic representation. Only RMTases involved in 2’-*O*-Me are indicated. Bottom, representation of an evolutionary shift in the dominance of stand-alone to snoRNP complexes from bacteria to plants. d) Arrow chart shows the number of Nm conserved in Arabidopsis [14,21], tomato [59] and tobacco [54] plants and the number of Nm equivalent in other species: human [1,79], yeast [80], algae [34], archaea [81] and bacteria [29]

In all domains of life 2’-*O*-Me has emerged as an abundant and ubiquitous RNA modification. Activity of RMTase results in 2’-*O*-Me by catalysis of a methyl group transfer from *S*-adenosylmethionine (SAM) to the 2’ hydroxyl group of a ribose residue, resulting in a methoxy group and *S*-adenosylhomocysteine (SAH) (Figure 1A). This modification is present in diverse RNAs, including rRNA, tRNA, mRNA and other small regulatory RNA such as: small interference RNA (siRNA) and microRNA (miRNA) (reviewed in[1]). Each of the four nucleotides can be 2’-*O*-methylated (N to Nm, where N is G, A, U or C) resulting in structural changes of the modified RNA. On the one hand, 2’-*O*-Me favours the A’-form RNA helix conformation, instead of the Z-form RNA, a left-handed conformation for the RNA double helix, favoured by a sequence composed of purine/pyrimidine repeats and especially CG-repeats [2]. Furthermore, we 2’-*O*-Me stabilizes alternative secondary structures in which the Nm-modified nucleotides are paired [3]. On the other hand, 2’-*O*-Me might stabilize rRNA-mRNA, rRNA-tRNA or rRNA-protein interactions [2,4]. Moreover, resistance of 2’-*O*-methylated RNA nucleotides to alkaline and enzymatic hydrolysis has allowed its detection and localization on various RNAs via high-throughput methods (reviewed in [5]).

## Molecular mechanisms of C/D box snoRNP methylation

In yeast, archaea, plants and humans 2’-*O*-Me of rRNA, some snRNA and snoRNA, are guided by small nucleolar RNAs[84] (snoRNAs), called C/D-box snoRNAs (C/D snoRNAs) (Figure 1B). The C box (5'RUGAUGA3’) and D box (5'CUGA3’) of C/D snoRNAs are short consensus sequences that localize a few nucleotides away from the 5’- and 3’-ends, respectively. In the central part, the C/D snoRNA might contain also less conserved C’ and D’ motifs. One or two sequences localize upstream of the D or D’ box. They are about 10–21 nucleotides long and complementary to the rRNA sequence overlapping the site of 2’-*O*-Me. The rRNA nucleotide to be methylated is located precisely at the fifth position upstream from the D or D’ box (Figure 1B) [6,7].

To guide methylation, the C/D snoRNAs interact with proteins to form small nucleolar ribonucleoprotein (snoRNP) complexes, including the SAM-binding domain containing methyltransferase Nop1/Nop1p/fibrillarin (in archaea/yeast/in mammals), Nop5/Nop56p/NOP56, Nop58p/NOP58 and L7Ae/Snu13/L7Ae ([1,7–9] and Figure 1B).

In contrast, the genome of *Arabidopsis thaliana* encodes two fibrillarin proteins named FIB1 and FIB2, two NOP56 and NOP58 and four potential L7Ae genes [10–12]. The methyltransferase fibrillarin consists of an N-terminal GAR domain involved in nuclear signalling, a spacer region and a methyltransferase domain. The latter contains an RNA binding domain for guide RNA binding and methylation, while the C-terminal α-helix region interacts with NOP56/58. In Arabidopsis, the two fibrillarin proteins have similar structures in the methyltransferase domain [13]. The overlay of the structures indicates that the main structural difference results from an angle changed for the exposure of the GAR domain. Interestingly, Arabidopsis FIB1 and/or FIB2 can interact not only with hundreds of C/D snoRNAs [14] but also with other small and long non-coding RNA (ncRNA), including viral, small nuclear and small interfering RNA [15,16]. In addition to its RNA 2’-*O*-Me activity, Arabidopsis FIB2 can also perform methylation of histone H2A [17], similar to human fibrillarin [18]

## Spotlights on RMTases: from stand-alone enzyme to RNA guided methylation

In *E. coli*, 2’-*O*-Me of 16S and 23S rRNA are catalysed by site-specific methyltransferases. Thus, the single methyltransferases RlmB, RlmM and RlmE (RlmJ) modify the ribose of G2251, C2498 and U2552 in the 23S rRNA, respectively (Figure 1C and Table 1), whereas two separate methyltransferases, RsmI and RsmH, are responsible for 2’-*O*-Me of 16S-C1402. Interestingly, a homolog of *E. coli* RlmE (RlmJ) was also found in the archaea *Haloarcula volcanii* and likely catalyses 2’-*O*-Me of U2587 in *Haloarcula marismortui*. Ribose methylation of its equivalent site in the mitochondrial 21S-Um2791 of *S. cerevisiae* is also performed by a site-specific RMTase Mrm2 [19]. Its counterpart in the cytoplasmic 28S-Um2921 is implemented by the site-specific RMTase Spb1 and/or by a snoRNP associated with the guide RNA SnR52 (Lapeyre and Purushothaman 2004). Spb1 is also responsible for the methylation of the neighbouring 28S-Gm2922 site. The methylation of the equivalent *E. coli* 23S-U2552 in human is performed by FTSJ3 [1,20]. The Arabidopsis 2′-*O*-Me sites 18S-Cm1645 and 25S-Gm2620, are the equivalents of *E. coli* 16S-Cm1402 and 23S-Gm2251, respectively, and 2’-*O*-Me reactions at these positions are guided and performed by C/D snoRNP complexes (Figures 1C and 2 as well as Table 1). Unlike those sites, no snoRNA guide has been reported for the Arabidopsis 25S-Um2922 (the equivalent of *E. coli* Um2552) [14,21].

**Table 1.** Rmtase and/or C/D snoRNP involved in methylation of conserved rRNA sites in bacteria (E. coli), Archaea (*H. marismortui*), yeast (*S. cerevisiae*), mammals (*H. sapiens*) and plants (*A. thaliana*.).

Bacteria (E. coli)		Archaea (H. marismortui)		Yeast (S. cerevisiae)		Mammals (H. sapiens)		*Arabidopsis thaliana*	
2’-O-Me	RMTase	2’-O-Me	RMTase	2’-O-Me	RMTase	2’-O-Me	RMTase	2’-O-Me	RMTase/snoRNA
16S-Cm1402	RsmI (YraL) and RsmH			18S-Cm1639		18S-Cm1703		18S-Cm1645	FIB1 or FIB2/At1gCDbox192 (AtU43)
23S-Gm2251	RlmB (YfjH)	23S-Gm1950	aFIB+ sRNA	28S-Gm2619		28S-Gm4196		25S-Gm2620	FIB1 or FIB2/At1gCDbox25.2; At1gCDbox25.2 (AtU3/AtsnoR35)
23S-Cm2498	RlmM (YgfE)								
23S-Um2552	RlmE (RlmJ or FtsJ)	23S-Um2587	RlmE	28S-Um2921	Spb1, SnR52-snoRNP	28S-Um4498	FTSJ3	25S-Um2922	At3g19130 and At4g19610/nd
		23S-Gm2588	RlmE?	28S-Gm2922	Spb1	28S-Gm4499	FTSJ3	25S-Gm2923	At3g19130 and At4g19610/nd

nd = not detected snoRNA. See text for references.

**Figure 2.**
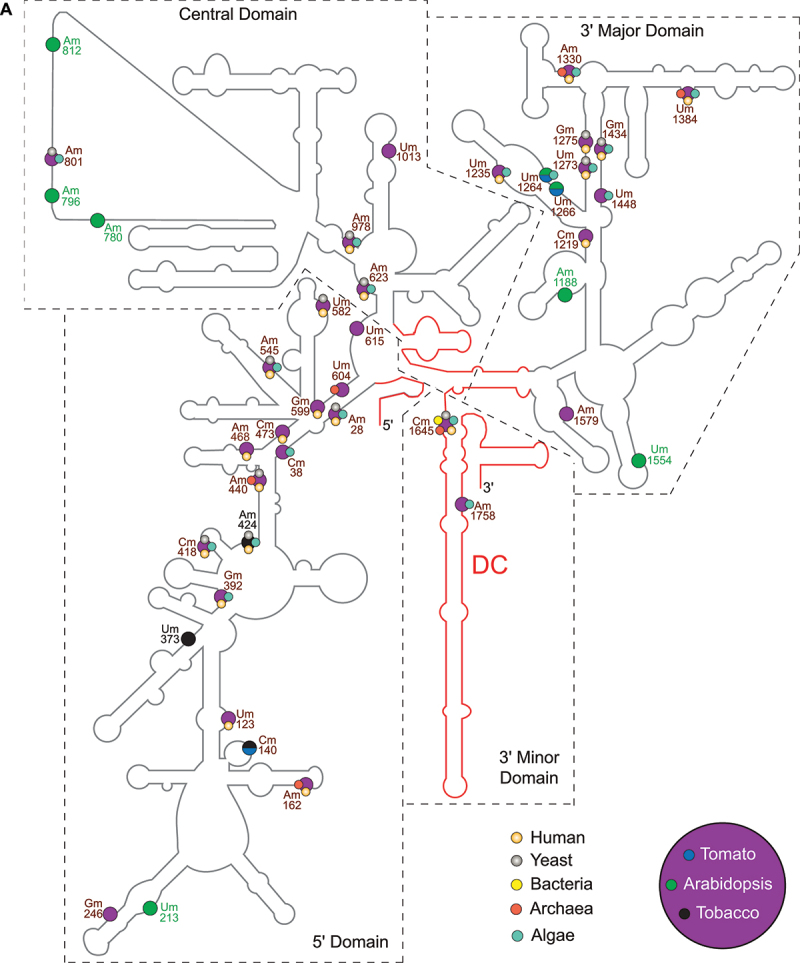
rRNA 2’-*O*-methylation sites (Nm) in a) 18S and b) 5.8S/25S cytoplasmic rRNA in plants (magenta), bacteria (yellow), archaea (turquoise) and human (orange) as well as specifically in Arabidopsis (green), tomato (blue) and tobacco (black) plants. Nm conserved in several or all species are labelled in brown, the species-specific are labelled as indicated before. The main function region of the rRNA are marked with red lines: DC (decoding centre; [82] in the 18S rRNA and PTC (peptidyl transferase centre); the intersubunit bridge [52] and GAC (GTPase associated centre; [83] in the 25S rRNA.

Like in yeast and human cells, most of the 118 rRNA Nm sites detected in Arabidopsis have corresponding C/D snoRNAs [14]. As a result of extensive gene duplications the Arabidopsis genome encodes 230 C/D snoRNAs with up to four of them targeting the same rRNA Nm [22–24].

Despite this plethora of C/D snoRNAs nearly 10% of the identified 2′-*O*-Me sites in Arabidopsis seem to lack a corresponding C/D snoRNA guide. Among them, five 2′-*O*-Me sites (Am812, Am1188 and Um1554 in the 18S and Um378 and Am2561 in the 25S) have been reported specifically in 21-day-old plants [14]. In contrast, the 25S-Um2922 (Um2552 in *E. coli*) and -Gm2923 are mapped in both 9- and 21-day-old plants, whereas the 25S 2′-*O*-Me sites Um676 is mapped only in 9-day-old plants [14,21]. Their lack of corresponding C/D snoRNAs hints towards an alternative guide mechanism or a stand-alone enzyme for these rRNA sites.

The Arabidopsis genome encodes three proteins without yet proven 2’-*O*-Me activity, but which are phylogenetically related to yeast Trm7p, Spb1p and Mrm2p. Noteworthy, the yeast Trm7p can 2’-*O*-methylate tRNA [19,25]. The determination of methylation at Nm sites without corresponding C/D snoRNA in Arabidopsis (Table 1) requires further investigation.

## Evolution of rRNA 2’-O-Me from bacteria to plants

Ribosomal RNA is the keystone of ribosome assembly and activity (reviewed in [26–30]). Among others, its biophysical properties are impacted by 2’-*O*-Me, which seems to be highly conserved in all kingdoms of life. In *E. coli*, 2’-*O*-Me can be found in four highly conserved nucleotides: in the 16S rRNA at position C1402 as well as in the 23S rRNA at positions G2251, C2498 and U2552 (Table 1). The 16S-Cm1402 participates in P-site (for peptidyl-tRNA) formation and seems to improve the precision of start codon selection, whereas the three Nm in the 23S are located in the Peptidyl Transfer Centre (PTC). Within the PTC, Gm2251 stays in close contact with the CCA-end of the P-site bound tRNA. U2552 is one of the conserved nucleotides in the A-site (for aminoacyl-tRNA) and interacts with incoming aminoacyl-tRNA (reviewed in [29]). In the archaea *H. marismortui*, all three detected rRNA Nm sites are located in the 23S rRNA (Table 1): at position G1950 (23S-Gm2251 in *E. coli*) as well as at the two neighbouring positions U2587 (23S-Um2552 in *E. coli*) and G2588 [31]. In archaea, the increased number of C/D snoRNAs was proposed to correlate with the increased growth temperature, which would necessitate 2’-*O*-methylation for the stabilization of rRNA folding [32].

There is clear evidence supporting the archaeal origin of eukaryotes. Archaea share 26 nucleotides signatures in ribosomal DNA with all living eukaryotes, no matter if protist, plant, fungus or animal [33]. However, 2’-*O*-Me profiles have evolved and display both conserved and kingdom-specific rRNA sites. In yeast, a total of 55 Nm is detected: 18 Nm in the 18S rRNA and 37 Nm in the 25S rRNA (sites conserved in plants are shown in Table 2 and Figure 2), but none in the 5S and 5.8S rRNA. In contrast, the 5.8S rRNAs of the unicellular marine alga and smallest photosynthetic eukaryote *Ostreococcus tauri* seems to contain two of the 101 Nm (sites conserved in plants are shown in Table 2). This prediction was based on genome annotated sequences of the C/D snoRNAs and locates the other rRNA Nm in the 18S (18 Nm) and 25S (29 Nm) rRNA [34].

**Table 2.** List of 2’-*O*-methylation sites experimentally mapped in *A. thalian*a [14,21], tomato [59], tobacco [54] plants and human [1,60], yeast [5], algae [34], archaea [62,63] and bacteria [29]. Arabidopsis sites marked with an asterisk indicate Nm sites for which no C/D snoRNA has been identified. np = non-mapped sites in *A*. *thaliana*, tomato, tobacco. For human, yeast, algae and archaea only Nm sites conserved in plants are listed.

rRNA	*A. thaliana*	Tomato	Tobacco	Human	Yeast	Algae	Archaea	Bacteria
		*(S. lycopersicum)*	*(N. tabacum)*	*(H. sapiens)*	*(S. cerevisiae)*	*(O. tauri)*	*(P. abyssi)*	*(E. coli)*
18S	Am28	Am28	Am28	Am27	Am28	Am28		
18S	Cm38	Cm38	Cm38			Cm38		
18S	Um123	Um123	Um123	Um121				
18S	np	Cm140	Cm140					
18S	Am162	Am162	Am162	Am166			Gm157	
18S	Um213	np	np					
18S	Gm246	Gm246	Gm246					
18S	np	np	Um373					
18S	Gm392	Gm392	Gm392	Gm436		Gm373		
18S	Cm418	Cm418	Cm418	Cm462	Cm414	Cm399		
18S	np	np	Am424	Am468	Am420	Am405		
18S	Am440	Am440	Am440	Am484	Am436		Am361	
18S	Am468	Am468	Am468	Am512				
18S	Cm473	Cm473	Cm473	Cm517				
18S	Am545	Am544	Am544	Am590	Am541	Am526		
18S	Um582	Am581	Um581	Um627	Um578			
18S	Gm599	Gm598	Gm598	Gm644				
18S	Um604	Um603	Ψm603				Gm512	
18S	Um615	Um614	Um614					
18S	Am623	Am622	Am622	Am668	Am619	Am604		
18S	Am780	np	np					
18S	Am796	np	np					
18S	Am801	Am800	Am800		Am796	Am770		
18S	Am812*	np	np					
18S	Am978	Am977	Am977	Am1031	Am974	Am946		
18S	Um1013	Um1012	Um1012					
18S	Am1188*	np	np					
18S	Cm1219	Cm1218	Cm1218	Cm1272				
18S	Um1235	Um1234	Um1234	Um1288		Um1202		
18S	Um1264	Um1263	np			Um1231		
18S	Um1266	Um1265	np					
18S	Um1273	Um1272	Um1272	Ψm1326	Um1269	Um1240		
18S	Gm1275	Gm1274	Gm1274	Gm1328	Gm1271			
18S	Am1330	Am1329	Am1329	Am1383		Am1297	Gm1064	
18S	Um1384	Um1383	Um1383	Um1442		Um1348	Um1115	
18S	Gm1434	Gm1433	Gm1433	Gm1490	Gm1428	Gm1398		
18S	Um1448	Um1447	Um1447			Um1412		
18S	Um1554*	np	np					
18S	Am1579	Am1579	Am1579					
18S	Cm1645	Cm1645	Cm1645	Cm1703	Cm1639	Cm1609	Cm1369	Cm1402
18S	Am1758	Am1758	Am1758			Am1720		
5.8S	Am47	Am48	Am46			Am42		
5.8S	Gm79	Gm80	Gm78	Gm75				
25S	np	np	Cm40					
25S	Um44	Um44	Um44					
25S	Um48	Um48	Um48			Um46		
25S	Um144	Um144	Um144			Um142		
25S	np	Am369	Am369					
25S	Um378*	np	Um378					
25S	Gm399	Gm399	Gm399					
25S	Am661	Am660	Am660	Am1326	Am649	Am557		
25S	Cm675	Cm674	Cm674	Cm1340	Cm663			
25S	Um676	Um675	Um675					
25S	np	np	Um787					
25S	Um803	Um804	Um804					
25S	Gm814	Gm815	Gm815	Gm1522	Gm805		Gm809	
25S	Am816	Am817	Am817	Am1524	Am807	Am694		
25S	Am826	Am827	Am827	Am1534	Am817	Am704		
25S	Am885	Am886	Am886		Am876		Am881	
25S	Gm917	Gm918	Gm918	Gm1625	Gm908			
25S	Am945	Am946	Am946				Am941	
25S	Um1067	Um1068	Um1068					
25S	Am1143	Am1144	Am1144	Am1871	Am1133	Am1015		
25S	np	Am1252	np					
25S	Am1263	Am1264	np			Am1135		
25S	Um1278	Um1279	np				Cm1233	
25S	Am1377	Am1378	Am1378			Am1259		
25S	Cm1447	Cm1448	Cm1448	Cm2351	Cm1437	Cm1319		
25S	Am1459	Am1460	Am1460	Am2363	Am1449	Am1331		
25S	Gm1460*	Gm1461	Cm1461	Gm2364	Gm1450			
25S	Cm1479	Cm1480	Cm1480					
25S	Cm1518	np	np	Cm2422				
25S	np	Um1537	Um1537					
25S	Cm1847	Cm1849	Cm1849					
25S	Cm1850	Cm1852	Cm1852					
25S	Gm1855	Gm1857	Gm1857			Gm1731		
25S	Cm1860	Cm1862	Cm1862	Cm2804		Cm1736		
25S	Am1871	np	np	Am2815				
25S	Um1892	Um1894	Um1894	Um2837	Um1888			
25S	Um2114	Um2116	np			Um1955		
25S	np	Gm2126	Gm2126			Gm1965	Gm1905	
25S	Gm2125	Gm2127	Gm2127					
25S	Am2127	Am2129	Am2129			Am1968		
25S	Cm2198	Cm2200	Cm2200	Cm3701	Cm2197			
25S	Am2215	np	np	Am3718				
25S	Am2221	Am2223	Am2223	Am3724	Am2220	Am2063		
25S	Gm2237	Gm2239	Gm2239			Gm2079		
25S	Am2257	Am2259	Am2259	Am3760	Am2256	Am2099		
25S	Am2282	Am2284	Am2284	Am3785	Am2281			
25S	Gm2289	Gm2291	Gm2291	Gm3792	Gm2288	Gm2131		
25S	Cm2294*	Cm2296	Cm2296			Cm2136		
25S	Am2322	Am2324	Am2324	Am3825				
25S	Am2327	Am2329	Am2329	Am3830			Gm2109	
25S	Cm2338	Cm2340	Cm2340	Cm3841	Cm2337	Cm2180	Cm2120	
25S	np	Um2350	Um2350		Um2347			
25S	Am2362	np	np	Am3867				
25S	Cm2366	Cm2368	Cm2368	Cm3869		Cm2208		
25S	Gm2392	Gm2394	Gm2394					
25S	Gm2396*	Gm2398	Gm2398	Gm3899				
25S	Gm2410*	Gm2412	Gm2412			Gm2252		
25S	Um2411	Um2413	Um2413					
25S	Um2422	Um2424	Um2424	Um3925	Um2421	Um2264		
25S	Um2456	Um2458	np					
25S	np	Gm2486	np	Gm4042				
25S	np	Cm2497	np	Cm4054				
25S	Um2494	np	np					
25S	Am2561*	np	np					
25S	Gm2620	Gm2623	Gm2622	Gm4196	Gm2619	Gm2451		Gm2251
25S	Am2641	Am2644	Am2643		Am2640	Am2472		
25S	Um2651	Um2654	Ψm2653	Um4227				
25S	Gm2652*	Gm2655	Gm2654	Gm4228		Gm2483		
25S	Cm2683	Cm2686	Cm2685				Cm2429	
25S	np	Um2721	Um2720					
25S	np	np	Um2732	Um4306	Um2729			
25S	Um2736	Um2739	Um2738					
25S	Gm2792	Gm2795	Gm2794		Gm2791	Gm2623		
25S	Gm2794	Gm2797	Gm2796	Gm4370	Gm2793			
25S	Gm2816	Gm2819	Gm2818	Gm4392	Gm2815	Gm2647		
25S	Cm2837	Cm2840	Cm2839			Cm2668		
25S	Cm2880	Cm2883	Cm2882	Cm4456		Cm2711		
25S	Um2884	Um2887	Um2886					
25S	Am2912	Am2915	Am2914					
25S	Gm2918	Gm2921	Gm2920	Gm4494				
25S	Um2922*	Um2925	Um2924	Um4498	Um2921		Um2669	Um2552
25S	Gm2923*	Gm2926	Gm2925	Gm4499	Gm2922			
25S	Am2935	np	np					
25S	Am2947	Am2950	Am2949	Am4523	Am2946			
25S	Cm2949	Cm2952	Cm2951		Cm2948			
25S	Um2954	np	np					
25S	Cm2960	Cm2963	Cm2962	Cm4536	Cm2959			
25S	np	np	Um3289					
25S	Gm3292	np	np					
25S	Um3301	Um3305	Um3304					

Arabidopsis sites marked with an asterisk indicate Nm sites for which no C/D snoRNA has been identified. np = non-mapped sites in *A. thaliana*, tomato, tobacco. For human, yeast, algae and archaea only Nm sites conserved in plants are listed.

Interestingly, higher eukaryotic cells have more Nm than single-cell organisms. For instance, in human and mouse rRNAs, up to 110 Nm sites (41 in 18S rRNA, 67 in the 28S rRNA and 2 in the 5.8S rRNA) have been detected so far ([35,36] and reviewed in [1,37] and Table 2).

In plants, the prediction of hundreds of potential rRNA 2′-*O*-Me sites relied majorly on bioinformatic analyses screening C/D snoRNAs encoding genes for short complementary rRNA sequences. However, only few of them have been experimentally verified in Arabidopsis and rice [12,37,and reviewed in 38,39]. Currently, the development of sequencing-based profiling methods allows the mapping of rRNA 2′-*O*-Me sites with and without annotated C/D snoRNAs [5,40,41]. Using RiboMethSeq, two independent studies mapped a total of 118 Nm sites in 9- and 21-day-old Arabidopsis plants [14,21]. Compared with RiboMethSeq from human and yeast, 51 rRNA 2′-*O*-Me sites seem to be Arabidopsis-specific, while a subset of 36 Nm is conserved in both yeast and human; only 5 Nm sites were conserved only in yeast and 28 Nm only in human (Figure 2 and Table 2).

**Figure 2.**
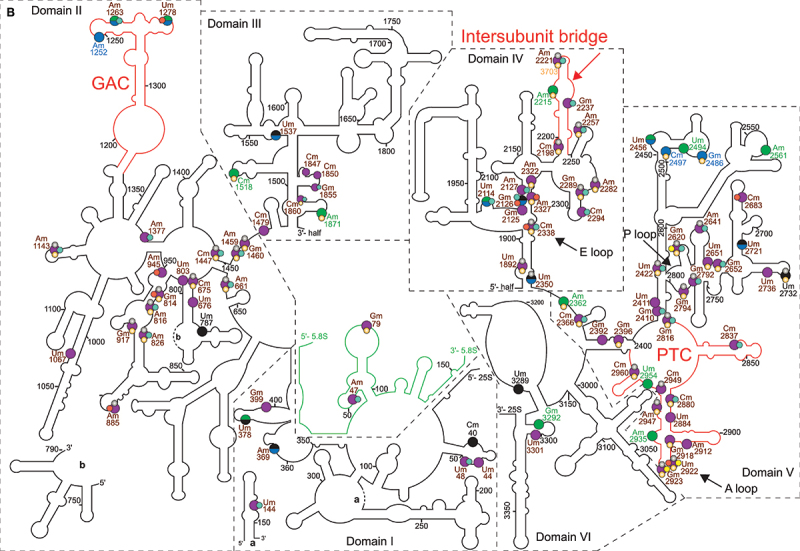
(continued).

Altogether the number of rRNA sites mapped in Arabidopsis by RiboMethSeq (up to 38 Nm in the 18S, 2 Nm in the 5.8S and up to 77 Nm in the 25S) is much lower compared to the 212 (78 Nm in the 18S, 3 Nm in the 5.8S and 131 Nm in the 25S) sites annotated as methylated or potentially methylated [39]. Whether or not these sites are 2’-*O*-methylated under specific plant growth conditions, development stages or in response to environmental stress remains to be investigated.

## Regulating rRNA Nm during plant growth and development

Expression and assembly of the C/D snoRNP components have been associated with plant development in Arabidopsis. Knockout of C/D snoRNAs HIDDEN TREASURE 2 (HID2) or SnoR28.1, triggers strong developmental and growth defects, which are even more pronounced for the double mutant [42,43].

Mutant plants for HID2 and SnoR28.1 exhibited pleiotropic developmental defects, including delayed seed germination, retarded root growth, and narrow, pointed leaves at the adult stage, and delayed transition to the reproductive phase was also observed in SnoR28.1 mutant plants. At the molecular level, knockout of SnoR28.1 appears to affect both methylation of the rRNA predicted target site and, to a less extent, pre-rRNA processing, whereas knockout of HID2 only affects pre-rRNA processing [42,43]. Furthermore, gene disruption of NUFIP, a C/D snoRNP assembly factor, inhibits 2’-*O*-Me at specific rRNA sites and leads to severe developmental phenotypes [44]. For instance *nufip* plants showed significant growth delay as compared with wild type plants or had premature growth arrest and did not reach the adult state. The *nufip* seedlings had pointed leaf phenotype, phyllotaxy defect, floral defects, reduced fertility or simply no seeds and sterility [44].

Despite that, no major profile changes were observed when comparing 9- or 21-day-old Arabidopsis plants [14,21]. Only a few Nm detected in specific growth or developmental conditions. For instance, the 25S-Am1871 and 25S-Um2954 sites were reported only in 21-day-old plants. All these sites have assigned C/D snoRNAs and, therefore, the methylation dissimilarities could be due to differential C/D snoRNP expression (of C/D snoRNAs or core proteins) or assembly. Similarly, 18S-Um1554 was detected in 21-day-old [14], but not in 9-day-old plants [21], while the 25S-Um676 was detected in 9-day-old plants but not in 21-day-old plants [21]. However, these sites do not have assigned C/D snoRNAs [14] and the methylation dissimilarities could be due to differential expression of a potential stand-alone RMTase. Besides differential expression of C/D snoRNAs and core proteins or stand-alone RMTases; modifications and turnover of proteins and C/D snoRNAs may also control 2’-*O*-Me activity. In particular, in human N6-methylation of adenine, was shown to disrupt K-turn formation and thus binding of the C/D snoRNP core protein15.5 kD [45]. In addition, snoRNP-associated factors may also affect 2’-*O*-Me activity (reviewed in [46]).

## Ribosome 2’-O-Me

In yeast and animal cells, 2’-*O*-Me of rRNAs occurs during transcription of 45S rRNA, the precursor of the 18S, 5.8S and 25S rRNAs. The transcribed 45S pre-RNA is then subjected to a number of co-transcriptional or post-transcriptional cleavages and assembly reactions with ribosomal proteins to form 40S and 60S ribosomal particles (reviewed in [10,27,47]). Formation of ribosomes and translation is initiated, when the initiator tRNA carrying methionine (tRNA^me^) attaches to the 40S ribosomal subunit. The 40S-tRNA^met^ complex interacts with the 5’-end of the mRNA, by recognizing the 5’ GTP cap, scans to find the start codon (AUG) and allows binding tRNA^me^::AUG. Then the 40S-tRNA^met^::AUG is joined by the 60S ribosome subunit to complete the ribosomal structure, creating the decoding centre and peptidyl-transferase activity (PTC) containing ribosome binding sites A, P and the exit (E) site.

The current view is that rRNA 2’-*O*-Me maintain stable ribosome structure [36,48,49]. In Arabidopsis, ribosome turnover is relatively low, replacing the population every 3–4 days [50] compared to a few hours in yeast [51]. Interestingly, the majority of 2’-*O*-Me modifications occur in conserved rRNA regions. Clustering of 2’-*O*-Me in the central, major and minor domains of the 18S rRNA locate them in the decoding centre. Other clusters were identified in domain IV and V of the 25S rRNA, which are involved in PTC activity and tRNA binding. All in all, these data support the role of ribose methylation in translation [52].

Interestingly, nearly half of the rRNA Nm sites detected in Arabidopsis have not been reported in yeast or animal cells ([14,21] and Figure 2 and Table 2). These Arabidopsis-specific rRNA Nm are in the central, 5’ major and 3’ minor domains of the 18S, in the 5.8S and in all domains of the 25S rRNA (Figure 2). Notably, Arabidopsis (plant) specific Nm are mapped in the hairpin structures H24 (Um1013), H31 (Am1188), H34 (Gm1448) and H44 (Am1758) forming the decoding centre. The loss of rRNA modifications of the decoding centre impairs pre-rRNA processing and ribosome translation in yeast [4]. Similarly, the Arabidopsis-specific rRNA Nm are mapped in functional domains of the 25S. In domain I, the H24 (Um378) interacts with SRPs and in turn with specific sequences in nascent translating polypeptides [53]. In the domain II, the H38 (Um1067) is involved in the formation of the intersubunit bridge between the 60S and 40S and it is contacting the A-site bound tRNA [54,55]. Meanwhile, H43 (Am1260) and H44 (Um1278) form the conserved GTPase centre [56]. In the domain III, the H47 (Cm1479) is required for processing of pre-27SB and 27SA into 25S [57]. And in the domain IV, the H68 (Gm2237), is involved in the formation of the inter-subunit bridge between the 40S and 60S [55,58] and it contains two E-sites [33]. In contrast, 2’-*O*-Me is not detected in the ES27, which is essential for translational fidelity, regulating amino acid incorporation and preventing frameshift errors. ES27 is also a scaffold for the conserved methionine amino peptidase (MetAP) that removes, co-translationally the first methionine from the nascent polypeptide chain. Similarly, the conserved sarcin/ricin loop (S/R-loop), which enables proper binding of elongation factors, does not seem to be 2’-*O*-methylated either. An attack of the S/R-loop by the ribonuclease α-sarcin and the RNA N-glycosidase ricin inhibits translation [26,54,59].

## 2’-O-Me profiling in tobacco and tomato ribosomes

To the best of our knowledge, 2’-*O*-Me profiling using RiboMethSeq or any other sequencing-based profiling methods have not been reported for any plant species other than *A. thaliana*. However, Cryo-EM structure (2.2 Ȧ resolution) studies of 80S ribosomes located rRNAs Nm from tomato [59] and tobacco [54]. In tomato, a total of 110 rRNA Nm were detected in the 18S (32 Nm), in the 25S (76 Nm) and in the 5.8S (2 Nm). In tobacco, a total of 107 rRNA Nm were detected in in the 18S (32 Nm), in the 5.8S (2 Nm) and in the 25S (73 Nm) (Table 2 and Figure 2).

Remarkably, the tobacco 18S-Cm1645 and 25S-Gm2622 (the equivalents of 16S-Cm1402 and 23S-Gm2351 in *E. coli*) locate at the P-site next to the mRNA and P/E tRNA in the small ribosomal subunit and at the P-site in the large ribosomal subunit next to the CCA tail of the A/P tRNA, respectively (Figure 2). A structural role of 25S-Gm2796 in the P/E tRNA and 25S-Am2259/25S-Gm2818 in the A/P tRNA site was also observed [54]. Furthermore, Cryo-EM density map allowed to propose that Gm1857 and Cm1849 together with Am827 might shape the N-terminal region of the ribosomal protein eL37 and is involved in constructing the peptide exit tunnel. Similarly, Am886 and Cm2920 interacts with methylated ribosomal protein uL3, which is crucial for proper rRNA processing [59].

These structural studies show that most of the rRNA Nm identified in Arabidopsis are detected in tomato and tobacco (Table 2). Regarding the predicted sites in Arabidopsis, 15 Nm were only mapped in tobacco and/or tomato, while 16 Nm were detected only in Arabidopsis but not in tobacco or tomato. Noticeably, the tobacco/tomato specific Nm in the 18S rRNA are located only in the 5’ domain, while the 25S specific Nm are in all five domains (I–V). In contrast, the Arabidopsis specific Nm in the 18S are distributed in the 5’, central and 3’ major domains and the 25S specific Nm are located only in the domain V; and more specifically the Am2935 in the A loop. Whether or not this indicates some kind of plant specific 2’-*O*-Me of the 40S and 60S ribosome remains to be investigated.

Altogether, Arabidopsis, tobacco and tomato plants have a total of 97 conserved Nm sites. While other species share a similar number of Nm sites, their level of conservation is very variable compared to plants. The most intriguing example is Archaea, which share only 13 of their 93 Nm sites with plants. Even *Ostreococcus*, phylogenetically quite close to plants, shares only half of its Nm sites with them. Algae therefore show a similar number of conserved Nm sites as human cells but their positions are not always identical. Then, it would seem that eukaryotic ribosomes need a similar number of Nm for their general function but their location meets species-specific needs (Figure 1D and Table 2).

While bacteria and yeast share 75% of their Nm sites with them, only half of the Nm sites in human and algae are among these conserved sites. From these sites 56, 41, 45 13 are also found in Nm in bacteria and yeast are conserved in plants

## Ribosome 2’-O-Me: major players

Evidence of ribosome heterogeneity at the rRNA level has been reported in mammals [36,48]. Differential expression of C/D snoRNAs has been reported in blood serum and plasma [60], changes of rRNA ribose methylation have been observed in developing tissues in mice [36] and alterations of 2’-*O*-Me have been associated to diseases, mainly cancer and autoimmune syndromes, and linked to tumour suppressor p53 [61,62].

Under normal cell conditions, p53 binds the fibrillarin gene promoter sequence and diminishes its expression level. In cancer cells, loss of function of tumour suppressor p53 provokes high fibrillarin activity resulting in the production of ribosomes with modified 2’-*O*-Me profile. These modified ribosomes translate mRNA with a lower fidelity and increase internal ribosome entry site (IRES)-dependent translation initiation of key cancer genes [62]. In contrast, inhibition of fibrillarin gene expression induces a global decrease in 2’-*O*-Me of the pre-rRNA in HeLa cells [48,62–64]. Likewise, p53 interacts with and re-localizes nucleolin in response to stress conditions [65]. Nucleolin is a multifunctional nucleolar protein required for rDNA expression, processing of rRNA and assembly of ribosomes. Nucleolin is also involved in DNA repair, remodelling and organization (reviewed in [66–68])

The homologue of p53 in plants is ANAC02 [69]. Disruption of ANAC082 in plant mutants for ribosomal biogenesis factors restores plant growth and developmental phenotypes but not the rRNA processing defects. The impact of ANAC082 on fibrillarin or nucleolin gene expression remains unknown.

Fibrillarin interacts also with the nucleolin protein in a large nucleolin-U3 snoRNP complex, which is involved in the processing of the largest rRNA precursors in yeast, mammals and plants [70–72]. Nucleolin protein could also affect C/D snoRNP assembly and/or methylation activity. Interestingly, inactivation of the homolog of the yeast nucleolin (Nrs1) depletes snoRNPs from the Dense Fibrillar Component (DFC) in the nucleolus, provoking their accumulation in a nucleolar body [73]. Nucleolin directly binds pre-rRNAs and snoRNPs, and could, thus, facilitate interactions of snoRNPs with pre-rRNAs (reviewed in [7]). Finally, nucleolin might also stimulate IRES-dependent translation [74].

Knockout gene expression of Arabidopsis nucleolin or fibrillarin activities has been demonstrated to provoke hypomethylation [14,21]. However, only *nuc1* plants show plant growth and developmental phenotypes [75–77]. A particular phenotype for *fib1* or *fib2* plant mutants has not been observed under normal or standard plant growing conditions, likely due to FIB1 FIB2 redundancy [21]. Interestingly, the amount of hypomethylated rRNA sites is similar in *nuc1* and *fib1* (80 Nm and 75 Nm, respectively) but higher compared to the number of sites in *fib2* (38 Nm). Among these sites 18 Nm are *nuc1* and 6 Nm *fib1/2* specific. Strikingly, disruption of the three genes never results in an increase of any 2’-*O*-Me at any rRNA position [14,21]. Finally, disruption of FIB2 provokes pathogen infection resistance [16], while, overexpression of NUC1 induces salt resistance [78]. A link with ribosome activity or methylation of stress/pathogen responsive genes has not yet been demonstrated.

## Concluding remarks

Despite their sessile nature, plants are able to adapt dynamically to environmental stresses through proteome modulation, primarily via translation regulation. Ribosomal RNA modifications, particularly 2’-*O*-Me, are crucial for ribosome activity and/or stability across all kingdoms of life. The species-specific profiles indicate adaptation to translational demands. The quantity of Nm increases along the phylogenetic tree with a transition from site-specific methyltransferases to a single (or two in Arabidopsis) methyltransferase. It is of interest to consider that the latter system is more energy efficient and easier to coordinate with the growing number of Nm. The presence of redundancy in targeting C/D snoRNAs and distinct 2’-*O*-Me profiles in single mutant of fibrillarin methyltransferases suggest that these C/D snoRNP component have specific roles in stress response and development, emphasizing the dynamic nature of rRNA methylation. In this context, rRNA Nm were identified without a corresponding snoRNA, which warrants further investigation of their methylation mechanism. It would be of interest to determine whether this is merely redundancy, ensuring the methylation of essential Nm despite the presence of hundreds of C/D snoRNAs. Additionally, a single C/D snoRNA can contain two antisense sequences, which raises the question how two different sequences evolve in the same C/D snoRNA. Here, emerging connections between ribosome 2’-*O*-Me, major players such as fibrillarin and nucleolin are also highlighted. However, further exploration of their role in C/D snoRNP assembly, rRNA methylation and its impact on translation efficiency is needed.

## Data Availability

There is no unpublished data mentioned in this review.
